# Prevalence and factors associated with frailty in hospitalized older patients

**DOI:** 10.1186/s12877-020-01545-4

**Published:** 2020-04-19

**Authors:** Sonia Hammami, Amira Zarrouk, Cecile Piron, Ioana Almas, Nabil Sakly, Veronique Latteur

**Affiliations:** 1 Department of Internal Medicine CHU F Bourguiba Monastir, Geriatric unit, University Hospital F. Bourguiba, Monastir, Tunisia; 2 Biochemistry Laboratory, LR12ES05 LR-NAFS ‘Nutrition - Functional Food & Vascular Health’Faculty of Medicine, University of Monastir, Monastir, Tunisia; 3 Geriatric Department, GHdC, Charleroi, Belgium; 4 Biochemistry Laboratory, Faculty of Medicine of Sousse, Sousse, Tunisia; 5 Department of Immunology, CHU F Bourguiba, Monastir, Tunisia

**Keywords:** Frailty, Dementia, Polypharmacy, CRP, Nutrition

## Abstract

**Background:**

Frailty is a multidimensional syndrome that leads to an increase of an age-related disorder of several physiological systems, and cognitive abilities decline. The aim of this study was to evaluate the prevalence of frailty among older persons in Belgium and we examined the factors associated with frailty with a principal focus en cognitive, dietary status, and inflammatory parameters.

**Methods:**

A total of 124 participants (90 women, 34 men; age: mean ± SD: 85.9 ± 5.5 years) were studied, recruited from the Geriatrics department, Belgium. Nutritional, cognitive status and physical activity were assessed using Mini Mental State Examination score (MMSE), Mini Nutritional Assessment score (MNA), and Katz score, respectively. Frailty syndrome was evaluated using the modified Short Emergency Geriatric Assessment (SEGA) score. Medication and medical history were recorded. Analyzed biochemical parameters included C-reactive protein (CRP), complete blood count, blood creatinine, vitamin D level, and serum protein electrophoresis. According to SEGA score, participants were divided into non-frail (*n* = 19), frail (*n* = 25) and severely frail patients (*n* = 80).

**Results:**

The SEGA score was inversely correlated with MMSE, MNA and Katz score. SEGA.

score was negatively correlated to albumin levels (*r* = − 0.30; *p* < 0.001) and positively correlated to CRP, polypharmacy and age (*r* = 0.28, *r* = 0.37, *r* = 0.33 and *p* < 0.01 respectively). Logistic regression showed a strong association between frailty, Katz score, dementia, polypharmacy and living in nursing home.

**Conclusion:**

Our results provide useful information for understanding mechanisms of frailty. This will help to develop preventive strategies for the elderly at the pre-frailty stage.

## Background

Frailty has been described as a clinical state functional reserve decline associated with aging. Slowness, weakness, exhaustion and low activity are combined and affect the performance of functional tasks negatively [1]. Disability, hospitalization, fragility, fracture, institutionalization, and early mortality are the major frailty consequences [2, 3]. Several physiological systems are dysregulated in frailty and lead eventually to function loss; such as musculoskeletal functioning, the inflammatory system, and the endocrine system [4–6]. Frailty can be considered as a complex phenomenon, some studies proposed that frailty can be defined as an at risk state caused by the age-associated accumulation of deficits. This accumulation model suggests that the more deficits individuals accumulate, the more they are at risk of an adverse health outcome [5, 6].

According to several studies, frailty is associated with cognitive impairment. In fact, cognitive impairment or dementia is a clinical syndrome that manifests as a decline in cognition, attention, memory, and language that are able to impair daily living activities of a person [7].

It has been reported that severe cognitive decline were accompanied by decreased physical function, poor muscle strength, slow gait speed, and weight loss; which ultimately results in the affected elderly being completely dependent on others [8, 9]. In addition, associations between slower Timed up and go (TUG), poorer executive function and global cognitive impairment have been reported [10]. Hence, a new term combining physical frailty with cognitive impairment was defined by the International Consensus Group as «cognitive frailty». Thus, a new possibility to characterize the relation between the two impairments and to detect elders at-risk with cognitive impairment caused by non-neurodegenerative conditions and then to develop interventions, improving their life quality, is now provided. In literature, some indicators of frailty have been shown better predictor of cognitive decline than others. Boyle et al. (2010) suggested that grip strength and timed walk are important indicators of the Mild Cognitive Impairment (MCI) diagnosis [11]. Previous studies reported poorer mobility in groups with poor cognitive function [12, 13] and associations between slower TUG and poorer executive function and memory [14, 15]. Recently, it has been reported that slow gait speed in older adults with MCI was associated with polypharmacy [16]. Polypharmacy is defined as the combination of five or more medications. It has attracted considerable interest in the field of geriatric medicine. The number of medications has been associated to an increased risk of adverse outcomes. The unfavorable consequences of polypharmacy might be explained by poor adherence that makes it difficult to attend the wanted clinical goals or by drug-drug interactions which could increase adverse drug reactions.

The aim of this study was to analyze the relationships between frailty and dementia in a sample of Belgian elderly. Furthermore, we intended to evaluate the causal link between biochemical measures and frailty, with a special focus on inflammation and nutrition.

## Methods

### Patients

This is a retrospective cohort study that used data from the department of geriatric, GHdC Belgium in the period between January and March 2018. Our analysis focused on 124 patients (90 women, 34 men; age: mean ± SD: 85.9 ± 5.5 years), who are selected according a random sampling process. Protected health information was scrubbed from both structured and unstructured data prior to the analysis. All data was stored on a secured network approved by the institution.

Patients’ information regarding age, gender, residency, medical history, number of drugs used, and laboratory evaluation was recorded. Patients who were younger than 65 years, with medical emergency or severe dementia, unable to communicate, and who do not consent were excluded from the study. Data were collected through questionnaires including medical history, clinical examinations, geriatric assessment and laboratory analysis. Weight and height were measured using standard techniques. Body Mass Index (BMI) was calculated as weight (kg) divided by height (m) squared. All the patients underwent comprehensive geriatric assessment. Depression, was assessed using the Geriatric Depression Scale (GDS-15) and defined as having a score of > 4 [17]. Mini-Mental State Examination (MMSE) was used to evaluate the cognitive function, using the cutoff point < 22 [18]. Mini Nutritional Assessment (MNA) was used to evaluate nutrition, using the cutoff of 17 [19]. The frailty status of these patients was evaluated using the modified version of the modified Short Emergency Geriatric Assessment (SEGA m) Validated in 2014 by the SFGG. The maximum score is 26 points, representing the highest level of frailty. Individuals scoring from 0 to 8 points are considered “non frail”, 9 to 11 “frail” and 12 points or more “severe frail”. Independence in Activities of Daily Living (ADL) was assessed using the Katz Score. The Katz score consists of six item (bathing, dressing, toileting, transferring, continence and feeding); each item was scored as dependent vs independent. The total sum score ranges from 0 (dependent) to 6 (independent). Dependency was defined as deterioration on at least one domain of ADL (score < 6) [20]. Polypharmacy is stated as concomitant five or more drug usage. Venous blood samples collected from each participant were subject to laboratory tests. Assays were performed according to the manufacture’s protocols. Conventional biochemical characteristics of elderly patients including vitamin D level, serum protein electrophoresis, glycaemia, glycated hemoglobin, a complete blood count, blood creatinine, C Reactive Protein (CRP), and albumin were realized.

### Statistical analyses

Results are expressed for continuous variables as the mean ± standard deviation and for qualitative variables as frequencies. Analyses were carried out by the Mann-Whitney U test or by the one-way analysis of variance (ANOVA) and Duncan’s multiple range test with SPSS version 22 (Statistical Package for Social Science, SPSS Inc., Chicago, IL). The Spearman correlation test was also used to evaluate the relationships between various parameters. Data were considered statistically different at a *p*-value of 0.05 or less. Logistic regression was performed to assess the relationship between frailty status and predictor variables. The presence of severe frailty according SEGA score was the dependent variable.

## Results

**Fig. 1:**
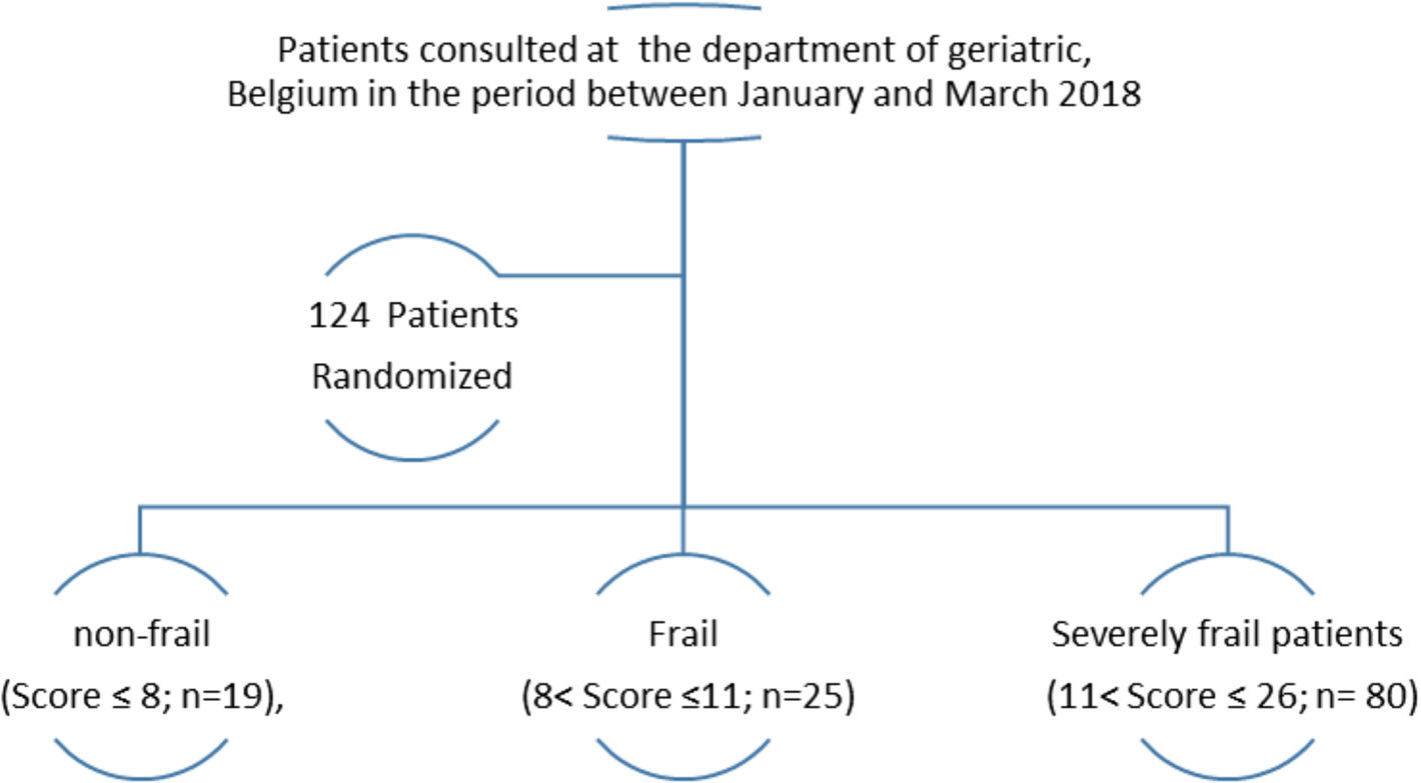
Flow diagram of the participants

The population includes 124 participants older than 65 years. The demographic features of the study population are summarized in Table 1. These participants were distributed, according to SEGA score, into non-frail (Score ≤ 8; *n* = 19), frail (8 < Score ≤ 11; *n* = 25) and severely frail patients (11 < Score ≤ 26; *n* = 80) Fig. 1. Characteristics of the participants categorized by frail status are described in Table 2.

**Table 1:** Baseline characteristics of the study population

		Total ***n*** = 124 (%)
**Age (years),Mean ± SD** **Age ≥ 85 years**		85.9 ± 5.5 78 (63)
**Gender (Female/Male)**		90/34
**BMI (Kg/m**^**2**^**),Mean ± SD**		24.7 ± 5.7
**Residency**	Live alone	46 (37.1)
	With spouse and/or children	40 (32.3)
	Nursing Home	38 (30.6)
**Medical history**	Diabetes	26 (21.0)
	HTA	74 (59.7)
	Dislipoproteinemia	21 (16.9)
	Dementia	49(39.5)
	Depression	26 (21)
	confusion	59 (48)
	Cardiopathy	67 (54.0)
	Osteoarthritis/ osteoporosis	123 (99.2)
	Incontinence	39 (31.7)
	Falls	60 (48.4)
**Professional medical frame**		90 (72.6)
**Polypharmacy ≥ 5**		88 (71)
**Length of hospitalization (days)**		20.0 ± 15.1
**Comprehensive geriatric assessment**	MMSE Score	17.7 ± 6.8
	Dependency for ADL Katz score < 6	104 (84.6)
	Malnourished MNA < 17	60 (50.4)
	SEGA score	12.9 ± 3.9
**Disease progression**	Amelioration	47 (38.2)
	Stabilization	55 (44.7)
	Death	21 (17.1)

*SD* Standard deviation, *BMI* Body Mass Index, *ADL* Activities of Daily Living, *MNA* Mini Nutritional Assessment, *SEGAm* modified Short Emergency Geriatric Assessment

**Table 2:** Differences regarding characteristics of frail and non-frail subjects

Characteristics		Non frail ***n*** = 19(%)	Frail ***n*** = 25(%)	Severely frail ***n*** = 80(%)
**Age (years),Mean ± SD** **Age ≥ 85 years**		83.2 ± 7.2 8 (42)	85.4 ± 4.8 16 (64)	86.8 ± 5.1 ^*^ 53 (67.5)*
**Gender (Female/Male)**		12/7	21/4	57/23
**BMI (Kg/m**^**2**^**),Mean ± SD**		25.8 ± 4.8	25.3 ± 5.7	24.3 ± 5.9
**Residency**	**Live alone**	11 (57.9)	17 (68)	18 (22.5)
	**With spouse and/or children**	8 (42)	6 (24)	26 (32.5)
	**Nursing Home**	0	2 (8)	36 (29.0)
**Medical history**	**Diabetes**	4 (21)	7 (28)	15 (19)
	**HTA**	9 (47.4)	16 (64)	49 (61.3)
	**Dislipoproteinemia**	4 (21)	5 (20)	12 (15)
	**Dementia**	4 (21)	5 (20)	40 (50)*
	**Depression**	4 (21)	6 (24)	16 (20)
	**Confusion**	4 (21)	10 (40)	45 (57)*
	**Cardiopathy**	7 (36.8)	17 (68)	43 (53.8)
	**osteoarthritis /osteoporosis**	19 (100)	25 (100)	79 (98.8)
	**Falls**	6 (31.5)	13 (52)	41 (51.2)
	**Incontinence**	1 (5.3)	6 (24)	32 (40.5)*
**Polypharmacy ≥ 5**		7 (36.8)	18 (72)	63 (78.8)*
**Disease progression**	**Amelioration**	11 (57.9)	12 (48)	24 (30.4)
	**Stabilization**	4 (21)	10 (40)	41 (51.9)
	**Death**	4 (21)	3 (12)	14 (17.7)
**Length of hospitalization (days)**		18.8 ± 20.9	21.6 ± 10.8	19.7 ± 14.8
**Comprehensive geriatric assessment**	MMSE Score	23.2 ± 5.8	20.1 ± 6.1	15.6 ± 6.2 *
	Dependency for ADL Katz score < 6	2 (10.5)	15 (60)	77 (96.3)*
	Malnourished MNA < 17	5 (27.8)	7 (29.2)	48 (62.3)*
	SEGA score	6.7 ± 1.5	10.4 ± 0.8	15.2 ± 2.6 ^*^
**Professional medical frame**		3 (15.8)	16 (64)	71 (88.8)

Significant difference between values at *p* < 0.05 level is indicated by *. *SD* Standard deviation, *BMI* Body Mass Index, *ADL* Activities of Daily Living, *MNA* Mini Nutritional Assessment, *SEGAm* modified Short Emergency Geriatric Assessment

Of the total patients, 90 (72.6%) were women, yielding a male-female ratio of 0.37. A statistically significant age difference was observed (*p* < 0.05).The mean age of the subjects was 85.9 ± 5.5 years, with a higher prevalence of subjects aged over 85 years old among severely frail patients compared to the others groups. The Body Mass Index (BMI) of the studied population was in the normal weight range with a mean of 24.7 ± 5.7 kg/m^2^. However, BMI was slightly higher than 25 for non-frail and frail groups. The percentages of residing in nursing home or living with a spouse and /or children were higher for severely frail patients (29.0 and 32.5%, respectively) than the frail and non-frail patients. Almost all the subjects had gonarthrosis or osteoporosis (99.2%), and above half of the subjects reported their initial diagnosis as cardiac disorder and hypertension (54.0 and 59.7% of total patients, respectively) with severely frail being the most represented (53.8 and 61.3%, respectively). Dementia, confusion and incontinence were all more common (*p* < 0.05) in severely frail than frail and nonfrail groups (Table 2). The majority of the studied patients (71.0%) and in particular the severely frail group (78.8%) consume more than 5 drugs per day (Tables 1 and 2).

Cognitive and frailty screening were conducted using MMSE and SEGA tests. Severely frail patients had the significantly lowest MMSE score (*p* < 0.05). The mean Katz score was 2.8 in all frail patients and 84.6% had a Katz score less than 6, indicating an impairment in daily living autonomy in these patients. The highest prevalence of dependency was observed in severe frail patients (96.3%). The Mini Nutritional Assessment (MNA) was performed in elderly patients and the mean score was of 16.2 ± 4.6 in total frail, indicating that most of these individuals are malnourished. Stratification of the study population according to the MNA score, showed that 50.4% were malnourished and had an MNA score lower than 17. The highest prevalence of malnourished patients was observed in severely frail patients (62.3%) (Table 2) and in patients with dementia (28.3% of total demented patients) (Data not shown). Biochemical parameters of frail subjects are summarized in Table 3. Compared to reference values, higher levels of CRP were observed in total frail patients. CRP and Vit D levels were significantly higher in severely frail patients than the other groups (*p* < 0.05). Whereas, lower levels of albumin and prealbumin were observed (Table 3).

**Table 3:** Biochemical characteristics and differences of frail and non-frail subjects

Population	Total ***n*** = 124	Non frail ***n*** = 19	Frail ***n*** = 25	Severely frail ***n*** = 80
**Glycemia (mg/dL)**	98.0 [32.0–661.0]	89.0 [53.0–241.0]	104.0 [60.0–661.0]	97.0 [32.0–271.0]
**HbA1c (%)**	6.8 ± 12	6.3 ± 1.3	6.5 ± 1.5	7.0 ± 1.1
**Cholesterol (mg/dL)**	163.0 ± 41.8	182.5 ± 50.8	163.5 ± 34.8	157.9 ± 40.2
**Leukocytes (10**^**3**^**/μL)**	9.0 ± 4.4	9.2 ± 3.2	8.2 ± 5.0	9.3 ± 4.5
**Lymphocytes (%)**	16.6 ± 9.6	15.2 ± 9.3 ^b^	20.8 ± 11.3 ^a^	15.6 ± 8.9 ^b^
**Hemoglobin (g/dL)**	12.1 ± 2.7	12.5 ± 1.8	11.8 ± 1.8	12.2 ± 3.1
**CRP (mg/L)**	30.5 [3.0–333.0]	19.0 [3.0–315.0]	12.0 [3.0–229.0]	44.5 [3.0–333.0] *
**Albumin (g/L)**	29.6 ± 5.1	31.6 ± 4.1	30.6 ± 6.3	28.8 ± 4.8
**Prealbumin (mg/dL)**	16.5 ± 6.8	19.1 ± 6.9	17.1 ± 6.7	15.7 ± 6.8
**Creatinin (mg/dl)**	1.1 ± 0.8	1.4 ± 1.0	1.0 ± 0.4	1.1 ± 0.8
**Vit D (ng/ml)**	21.6 ± 12.0	15.9 ± 9.7 ^b^	20.3 ± 12.5 ^a b^	23.4 ± 12.0 ^a^

*Vit D* Vitamin D, *CRP* C Reactive Protein, *HbA1c* Glycated hemoglobin

* Correlation is significant at the 0.05 level (Mann-Whitney test)

^a, b^: Different superscript letters in the same row indicate significant difference between values at p < 0.05 level (Duncan’s multiple range test) and values are mean ± standard deviation or or median [minimum; maximum] (non-normal distribution)

Among frail population, the SEGA score was negatively correlated to MMSE, MNA and Katz scores (*r* = − 0.50, *r* = − 0.39 and *r* = − 0.67; *p* < 0.01). Furthermore, SEGA score was negatively correlated to albumin levels (*r* = − 0.30; *p* < 0.01) and positively correlated to CRP, polypharmacy, and age (*r* = 0.28, *r* = 0.37, and *r* = 0.33, respectively; *p* < 0.01) (Table 4).

**Table 4:** Correlation Coefficients according to SEGA score for selected items

Variables	SEGA score
**Age**	0.33^a^
**MNA score**	−0.39^a^
**MMSE score**	*−0.50a*
**Katz score**	*−0.67a*
**Polypharmacy (number)**	0.37^a^
**CRP**	0.28^a^
**Albumin**	−0.30

*CRP* C Reactive Protein

*MMSE* Mini mental state examination

*MNA score* Mini Nutritional Assessment

^a^Correlation is significant at the 0.05 level

Using logistic regression, we found that dementia, polypharmacy ≥5, living in nursing home, and decrease of functional capacity evaluated by Katz score, were all associated with severe frailty as shown in (Table 5).

**Table 5:** Correlates of very frail among the study subjects (results of multiple logistic regression analysis)

Variable	β-Coefficient (SE)	Odds ratio (95% CI)	p
**Dementia**	1.39 (0.62)	4.04 *(1.1–4.7)*	0.02
**Polypharmacy** ***≥*** **5**	1.18 (0.59)	3.2 *(1.01–6.7)*	0.04
**Nursing home**	1.23 (0.47)	3.42 *(1.5–4.9)*	0.003
**Katz Score**	3.09 (0.72)	4.7*(1.9–5.6)*	0.000

Age, sex, depression, MNA score, CRP, Albumin, confusion were not statistically significant

*SE* standard errors, *CI* confidence interval

## Discussion

The global interest in the study of aging processes and age-related diseases is due to the rise in the elder’s proportion associated with an increased sanitary implication. Frailty constitutes a precise measurement of aging symptoms and it indicates a multidimensional syndrome of energy, physical ability, and cognition loss. This syndrome has been shown to be potentially preventable and could be reverted in its earlier stages. Thus, we conducted a retrospective study in Belgian elders (*n* = 124, aged 65 and over), classified according to their frailty status, in order to increase evidence related to frailty and to find parameters that could be used as early indicators.

The current study examined the relationship between frail status and cognitive function in Belgian elderly. We confirmed that physical frailty is correlated with a decline in cognitive functions, which support previous findings. Indeed, data from the Rush Memory and Aging study found that higher levels of frailty were associated with a faster rate of decline in all cognitive domains [11]. Furthermore, the results of Wu et al. (2015) indicated that the appearance of memory impairment may indicate its association with higher frail status, suggesting that existing cognitive impairment is a risk factor for an additional frail decline [21]. Also, it has been shown that cognitive function across all domains was significantly worse in frail participants than non-frail, with the exception of self-rated memory and processing speed. Weakness and walking speed were also linked to poorer cognition [22]. However, our findings contradict some studies suggesting the absence of an association between memory decline and frailty [8, 23, 24]. This discrepancy could be explained by the size or the homogeneity of the samples in these studies [8, 23, 24].

Biological and psychological factors, including neuropathology, cardiovascular disease, inflammation, hormonal changes, nutrition, social vulnerability, and isolation have been suggested to explain the link between frailty and cognition [25]. In the present study, we tried to find an explanation for this association. Thus, several biochemical measures, frail status assessments and neuropsychiatric assessment, including the Mini-Mental State Examination have been performed in a population of Belgian elderly patients.

Some biochemical measures were associated with frailty. In fact, frailty was associated with CRP and albumin levels. It is well known that serum albumin is the most abundant blood protein and used as a marker of nutritional status. Hypoalbuminemia can reflect complications in different systems in elderly subjects. Since frailty is related to dysfunction in several organs, that could explain the observed inverse association between albumin and frailty index in the study population. These data are in accordance with others studies demonstrating that low albumin concentrations were associated with higher frailty scores [26–28]. Recently, hypoalbuminemia was associated with chronic inflammation [29]. In fact, chronic low-grade inflammation, is considered as a risk factor for the development of aging-related diseases, has been found to be associated with organ damage, muscle waste and chronic diseases, which all contribute to frailty [7]. On the other hand, chronic inflammation appears as a consequence of chronic diseases such as atherosclerosis and Alzheimer dementia [30]. This phenomenon has been linked to both frailty and cognitive function [25]. Furthermore, several studies support the direct association between serum CRP levels and frailty in elders [31]. In accordance, we found that elevated levels of CRP were associated with higher frailty scores in the study population.

Hypoalbuminemia has also been used as a marker of malnutrition [29]. Hence, the observed correlation between frailty and albumin deficiency could reflect a poor nutritional status in the studied population, suggesting that malnutrition is associated with higher frailty. Nutritional deficiencies could reflect insufficient micronutrient intake. Knowledge about the relationship between micronutrient status and frailty could promote interventions to correct micronutrient deficiencies. Insufficient serum 25-hydroxyvitamin (25(OH)D) concentrations were associated with frailty status and measures of physical performance [30].

Contrary to the literature, we could not find an inverse correlation between Vitamin D and frailty score [6, 27, 32]. However, this is comparable to data of Schoufour et al. (2015) study,

conducted on elderly people with intellectual disabilities [28]. Furthermore, the Vitamin D levels were higher in frail and severely frail patients compared to non-frail. This could be explained by the supplementation since sufficient 25(OH)D was considered crucial for the frailty prevention. Recently, it has been reported that among the hospitalized elders without Vitamin D supplementation, Vitamin D deficiency was prevalent suggesting a necessity to supplement Vitamin D in order to maintain desirable levels [33].

In addition, the multivariable model using logistic regression identified dementia, polypharmacy ≥5, living in nursing home, and decrease of ADL as significantly asssociated to frailty (*P* < 0.05). Thus, our study confirms the existence of an association between the prevalence of frailty and the number of drugs prescribed. Indeed, previous studies indicated that frail patients were likely to receive a higher number of drugs than non-frail ones [34, 35]. Also, it was reported that each additional drug was associated with frailty with an odds ratio > 1 [34, 36, 37]. The enhancement of the interactions and adverse reactions associated with each additional prescription could explain the effect of multiple drugs intake on frailty.

Also, in the Umegaki et al. (2019) study, the number of medications was associated with gait independently from the prescription of potentially inappropriate medications and from the Charlson Comorbidity Index [16]. In addition, it has been shown that the effects of psychotropic medications could be implicated in the underlying mechanism of the association between polypharmacy and gait speed [38]. However, this effect is still too small to fully explain it. In other hand, it has been demonstrated that patients with dementia used a higher number of medications [39, 40]. However, others found no association between number of medications and dementia or even showed that patients with dementia use a lesser number of medications [41]. This controversy is not surprising because the effects of the medications type are diverse. Herr et al. (2015) suggested that polypharmacy may be utile to identify older patients, whose health is more susceptible to deteriorate and then to carry out corrective actions with regard to physical activity, nutrition, and management of chronic diseases [42].

Furthermore, it has been described that polypharmacy is common in the elderly and that residents nursing homes are taking the highest number of drugs [43]. Different studies have described higher prevalence of frailty in older adults living in nursing homes than in community based older adults [44, 45]. Recently, it has been shown that frailty but not pre-frailty was associated with increased nursing homes admission. Indeed, among community-living participants, those who were frail had a three times higher risk to be admitted to a nursing home, than those who were non-frail [46].

The knowledge of the factors associated to frailty represent target conditions for programs and policies directed at reducing frailty in older population [47]. Although, it is still unknown whether frailty risk accumulates or there is a required chain of events. It is also unknown if these factors identified precede or are the consequences of frailty. Indeed, the very complexity of the life course approach in the study of frailty should be considered to guide prevention actions. This would enhance the reliability of predictors factors of frailty in the early period (critical period) and could guide the preventives strategies.

However, our study has some limitations related to the small sample size and limited duration of observation. Indeed, the majority of the studied population was already in a severely frail state. Thus, recrutement of frail patients at an early state could be of interest, would enhance the reliability of predictor’s factors of frailty in the early period, and could allow the timely implementation of preventive strategies.

Furthermore, since frailty development is a life-long process, this study needs to be completed with repeated measurements and examination of frailty over time, which could give us new informations about the evolution of individual trajectories along with the different state of frailty. Also, proving the effect of a restriction of polypharmacy to its truly appropriate need or a dose reduction of medication is another approach to study the association between polypharmacy and frailty.

## Conclusion

Altogether, our data confirm the complicated pathophysiology behind frailty syndrome. Frailty associated parameters were given. We showed that dementia in particular Alzheimer disease, polypharmacy, malnutrition, and decrease on physical activity are risk factors for frailty development in older persons. The results are useful for identifying older individuals at risk of developing frailty and a new need for, enabling implementation of preventive strategies.

## Data Availability

The authors can confirm that all relevant data are included in the article.

## Abbreviations

BMI: The body mass index
CRP: C Reactive Protein
GDS-15: Geriatric Depression Scale
MMSE: Mini-Mental State Examination
MNA: Mini Nutritional Assessment
ADL: Activities of Daily Living
